# Geographic variation and racial disparities in adoption of newer glucose-lowering drugs with cardiovascular benefits among US Medicare beneficiaries with type 2 diabetes

**DOI:** 10.1371/journal.pone.0297208

**Published:** 2024-01-29

**Authors:** Wei-Han Chen, Yujia Li, Lanting Yang, John M. Allen, Hui Shao, William T. Donahoo, Lori Billelo, Xia Hu, Elizabeth A. Shenkman, Jiang Bian, Steven M. Smith, Jingchuan Guo

**Affiliations:** 1 Department of Pharmaceutical Outcomes & Policy, College of Pharmacy, University of Florida, Gainesville, Florida, United States of America; 2 Department of Pharmacy and Therapeutics, University of Pittsburgh, Pittsburgh, Pennsylvania, United States of America; 3 Department of Pharmacotherapy & Translational Research, College of Pharmacy, University of Florida, Gainesville, Florida, United States of America; 4 Center for Drug Evaluation and Safety (CoDES), College of Pharmacy, University of Florida, Gainesville, Florida, United States of America; 5 Hubert Department of Global Health, Rollin School of Public Health, Emory University, Atlanta, Georgia, United States of America; 6 Department of Family and Preventive Medicine, School of Medicine, Emory University, Atlanta, Georgia, United States of America; 7 Division of Endocrinology, Diabetes & Metabolism, Department of Medicine, College of Medicine, University of Florida, Gainesville, Florida, United States of America; 8 Office of Research Affairs, University of Florida College of Medicine-Jacksonville, Jacksonville, Florida, United States of America; 9 Department of Computer Science, Rice University, Houston, Texas, United States of America; 10 Department of Health Outcomes and Biomedical Informatics, College of Medicine, University of Florida, Gainesville, Florida, United States of America; Houston Methodist Academic Institute, UNITED STATES

## Abstract

**Background:**

Prior studies have shown disparities in the uptake of cardioprotective newer glucose-lowering drugs (GLDs), including sodium-glucose cotranwsporter-2 inhibitors (SGLT2i) and glucagon-like peptide-1 receptor agonists (GLP1a). This study aimed to characterize geographic variation in the initiation of newer GLDs and the geographic variation in the disparities in initiating these medications.

**Methods:**

Using 2017–2018 claims data from a 15% random nationwide sample of Medicare Part D beneficiaries, we identified individuals diagnosed with type 2 diabetes (T2D), who had ≥1 GLD prescriptions, and did not use SGLT2i or GLP1a in the year prior to the index date,1/1/2018. Patients were followed up for a year. The cohort was spatiotemporally linked to Dartmouth hospital-referral regions (HRRs), with each patient assigned to 1 of 306 HRRs. We performed multivariable Poisson regression to estimate adjusted initiation rates, and multivariable logistic regression to assess racial disparities in each HRR.

**Results:**

Among 795,469 individuals with T2D included in the analyses, the mean (SD) age was 73 (10) y, 53.3% were women, 12.2% were non-Hispanic Black, and 7.2% initiated a newer GLD in the follow-up year. In the adjusted model including clinical factors, compared to non-Hispanic White patients, non-Hispanic Black (initiation rate ratio, IRR [95% CI]: 0.66 [0.64–0.68]), American Indian/Alaska Native (0.74 [0.66–0.82]), Hispanic (0.85 [0.82–0.87]), and Asian/Pacific islander (0.94 [0.89–0.98]) patients were less likely to initiate newer GLDs. Significant geographic variation was observed across HRRs, with an initiation rate spanning 2.7%-13.6%.

**Conclusions:**

This study uncovered substantial geographic variation and the racial disparities in initiating newer GLDs.

## Introduction

Diabetes, the eighth leading cause of death in the US, affects almost 14% of US adults, and around 90–95% of them are diagnosed with type 2 diabetes (T2D).[1] T2D disproportionately affects racial and ethnic underrepresented groups (i.e., non-Hispanic Black, Hispanic, American Indian/Native Alaska, Asian/Pacific Islander) with higher disease prevalence, higher risks of diabetes complications, lower quality of care, and less access to glucose-lowering drugs (GLDs), compared to non-Hispanic White counterparts.[2] Few studies have recorded the geographic variation in T2D disease prevalence across the US.[3,4] However, no research has focused on the geographic variation of newer GLDs utilization.

With cardiovascular disease (CVD) being the leading cause of death among individuals with T2D, it is imperative that patients in need have sufficient and timely access to therapies and interventions to mitigate CVD risks.[5] Two newer therapeutic classes in GLDs, i.e., sodium-glucose cotransporter-2 inhibitors (SGLT2i) and glucagon-like peptide-1 receptor agonists (GLP1a) have been proven to improve both cardiovascular and renal outcomes in patients with T2D.[6] Specifically, among individuals with T2D, SGLT2i reduced the risks of major adverse cardiovascular events, hospitalization for heart failure, and chronic kidney disease;[7] GLP1a has demonstrated significant reductions in fatal or non-fatal stoke and myocardial infarction.[8] Current guidelines from American Diabetes Association have recommended the use of SGLT2i and GLP1a among T2D individuals at increased risk of and with established atherosclerotic CVD, heart failure, and chronic kidney disease (CKD), independent of glycemic status.[6]

Despite the clinical success, these newer agents remain under-used in the US and they may exacerbate the existing health disparities in T2D care and outcomes. Two recent studies found that, among commercially insured patients in the US, the use of SGLT2i and GLP1a therapies gradually increased from 2015–2019, but the overall use remained low (3.8%-11.9% and 3.2%-10.7%, respectively).[9,10] Furthermore, higher annual median household income is associated with 7%-13% higher initiation of newer GLDs. Both studies have also identified racial disparities in GLD initiation, whereby non-Hispanic Black, Hispanic, and Asian patients have lower initiation rates of newer GLDs compared to non-Hispanic White patients. A study of Medicare beneficiaries indicates that newer GLDs are increasingly adopted yet remain low and delayed, with 0.8% initiating SGLT2i and 1.0% initiating GLP1a.[11] In 2019, nearly 27.5% and 27.8% of Medicare beneficiaries had T2D and ischemic heart disease, respectively.[12] Nevertheless, initiation rates for these outcome-improving therapies among Medicare beneficiaries across the US remain unknown. Furthermore, racial and ethnic disparities have been well-documented in these newer GLDs,[13] but it is unclear whether and how such racial/ethnic disparities vary geographically across the nation.

In this study, therefore, we aim to document the initiation status of newer GLDs and the racial/ethnic disparities in a nationally representative sample of Medicare fee-for-service beneficiaries. Additionally, we characterize the geographic variation in the initiation of the newer GLDs, as well as geographic variation in GLD initiation disparities. Thus, our study may inform health policymakers where the most marginalized areas are so as to deliver interventions that are tailored locally, which may further improve access to better T2D treatment with cardiovascular benefits for racial/ethnic minority individuals with higher age and lower income.

## Methods

### Data sources and study population

In this cross-sectional study, we used administrative claims data from a 15% random sample of nationwide Medicare fee-for-service beneficiaries with Part D from January 1, 2017, to December 31, 2018, with oversampling of Florida that included 100% Florida fee-for-service beneficiaries. Medicare is a federally funded health insurance program covering individuals aged 65 or older across the United States. Over 98% of Americans aged 65 or older are enrolled in Medicare program. We employed a computable phenotyping algorithm that requires a T2D diagnosis (ICD-9 codes 250.x0, or 250.x2, or ICD-10 codes E11) and ≥1 antidiabetic prescription to identify individuals with T2D, which has been validated in claims data (PPV>96%).[14] We identified January 1, 2018 as the index date and included beneficiaries who did not use SGLT2i or GLP1a in the year prior to the index date (baseline year). We followed the study cohort for a year, from the index date through December 31, 2018. We excluded beneficiaries diagnosed with type 1 diabetes or who did not continuously enroll in Medicare Part D plan from January 1, 2017 through December 31, 2018 to ensure that the complete medical and prescription information was captured.

The study outcome was filling a prescription for newer GLD (GLP1a or SGLT2i) during the follow-up year or not. We collected beneficiaries’ sociodemographic and clinical information in the baseline year. Patient sociodemographic variables included age, sex, race/ethnicity (classified as non-Hispanic White, non-Hispanic Black, American Indian/Alaska Native, Hispanic ethnicity, Asian/Pacific Islander, and other using Research Triangle Institute [RTI] Race Code), rural/urban classification (defined using 2013 U.S. Department of Agriculture’s Rural-Urban Continuum Codes [RUCC]: 1–3 for urban and 4–9 for rural areas),[15] and Medicaid dual-eligibility. Clinical characteristics included T2D duration, number of GLD classes prescribed, history of CVD, and CKD. The cohort was spatiotemporally linked to Dartmouth hospital-referral regions (HRRs), with each patient assigned to 1 of 306 HRRs using the zip code.[16] The study was considered exempt by the Institutional Review Board at the University of Florida.

### Statistical analysis

We presented descriptive statistics for patients’ baseline characteristics by newer GLDs initiation status: mean (standard deviation [SD]) or median (interquartile range [IQR]) was computed for continuous variables, and frequency (percentage [%]) was calculated for categorical variables. Differences in baseline characteristics between the two groups were compared using Student *t*-tests and Pearson’s Chi-square tests for continuous and categorical variables, respectively. A *p*-value < .05 was considered statistically significant. All statistical analysis was conducted using SAS Enterprise Guide 8.3, and all map development was performed in ArcGIS Pro 2.7.0.

We built a multivariable Poisson regression model to determine racial and ethnic disparities in the initiation of newer GLD, with adjustment of sex, Medicaid dual eligibility, rural-urban classification, T2D duration, number of GLD classes prescribed, CVD, and CKD.

To examine geographic variation in newer GLDs initiation, we applied a multivariable Poisson regression model to estimate the adjusted initiation rate of newer GLDs across HRRs.[17] Covariates included age, sex, Medicaid dual eligibility, rural-urban classification, T2D duration, number of GLD classes prescribed, CVD, and CKD. We constructed the Poisson model and mapped the adjusted initiation rate of newer GLDs for the overall national cohort (15% samples of national fee-for-service beneficiaries) and for the cohort of Florida state (100% samples of Florida fee-for-service beneficiaries), respectively.

We further examined the geographic variation in racial disparities in newer GLDs initiation. We constructed a multivariable logistic regression model regressing newer GLDs initiation status against race/ethnicity, adjusting for age, sex, Medicaid dual eligibility, rural/urban classification, T2D duration, number of GLD classes prescribed, CVD, and CKD. The racial disparities (non-Hispanic Black vs. non-Hispanic White [referent]) in newer GLDs initiation status were identified across HRRs and categorized into four levels, corresponding to adjusted odds ratio (aOR) ≤0.5 (with *p* <0.05), >0.5 to ≤0.9 (with *p* <0.05), >0 to ≤0.9 (with *p* ≥0.05), >0.9 (regardless of *p*-value). We examined and mapped the disparities in newer GLDs initiation across HRRs for the overall national cohort and for the cohort of Florida, respectively. The disparities between the Hispanic vs. non-Hispanic White group were only calculated in Florida due to insufficient sample size for HRRs in the national cohort.

## Results

We identified 795,469 eligible Medicare beneficiaries with T2D who were treated with GLDs. Among this cohort, the mean (SD) age was 73.1 (10.5) years, 424,312 (53.3%) were women, 562,994 (70.8%) were non-Hispanic White, 96,891 (12.2%) were non-Hispanic Black, 84,744 (10.6%) were Hispanic, 29,645 (3.7%) were Asian/Pacific islander, and 4,621 (0.6%) were American Indian/Alaska Native. A total of 56,991 (7.2%) individuals initiated a newer GLD (i.e., an SGLT2i or GLP1a) in the follow-up year. Baseline patient demographics and clinical characteristics are summarized in Table 1. Compared to initiators of newer GLD, non-initiators were older (73.5 vs. 68.9 years), had longer T2D duration (7 vs. 6 years), were less likely to be non-Hispanic White (70.5% vs. 73.8%), and were more likely to have CVD (24.2% vs. 18.4%). Table 1. Baseline characteristics of Medicare beneficiaries with type 2 diabetes.

**Table 1:** Baseline characteristics of Medicare beneficiaries with T2D (N = 795 469).

Variable	Newer GLD Initiation		*P*
	No (N = 738 478)	Yes (N = 56 991)	
**Age, mean (SD), years**	73.5 (10.5)	68.9 (9.9)	< .001
**Sex, N (%)**			< .001
Male	342 932 (46.4)	28 225 (49.5)	
Female	395 546 (53.6)	28 766 (50.5)	
**Race/ethnicity, N (%)**			< .001
Non-Hispanic White	520 930 (70.5)	42 064 (73.8)	
Non-Hispanic Black	91 507 (12.4)	5 384 (9.5)	
Hispanic	79 033 (10.7)	5 711 (10.0)	
Asian/Pacific islander	27 670 (3.8)	1 975 (3.5)	
American Indian/ Alaska Native	4 306 (0.6)	315 (0.6)	
Other	15 032 (2.0)	1 542 (2.7)	
**Medicaid dual eligibility, N (%)**			< .001
No	486 365 (65.9)	35 223 (61.8)	
Yes	252 113 (34.1)	21 768 (38.2)	
**Rural/urban classification, N (%)**			< .001
Rural	139 482 (18.9)	11 380 (20.0)	
Urban	598 996 (81.1)	45 611 (80.0)	
**T2D duration, median (IQR), years**	7.0 (8.0)	6.0 (7.0)	< .001
**Number of GLD classes prescribed, median (IQR)**	1.0 (1.0)	2.0 (1.0)	< .001
**Cardiovascular disease, N (%)**			< .001
No	559 930 (75.8)	46 496 (81.6)	
Yes	178 548 (24.2)	10 495 (18.4)	
**Chronic kidney disease, N (%)**			< .001
No	254 745 (34.5)	12 156 (21.3)	
Yes	483 733 (65.5)	44 835 (78.7)	

GLD: Glucose-lowering drug; T2D: Type 2 diabetes; SD: Standard deviation; IQR: Interquartile range; *P*: *p*-value.

In the adjusted Poisson regression model for the overall cohort (Table 2), we observed that racial and ethnic minority groups (i.e., non-Hispanic Black, Hispanic, Asian/Pacific Islander, and American Indian/Alaska Native) were less likely to initiate a newer GLD than non-Hispanic White beneficiaries. Compared to the non-Hispanic White individuals, the disparities were the most distinct in non-Hispanic Black and American Indian/Alaska Native groups, which were associated with 34% (initiation rate ratio, IRR [95% CI]: 0.66 [0.64–0.68]) and 26% (IRR [95% CI]: 0.74 [0.66–0.82]) lower rates of new GLD initiation, followed by Hispanic (IRR [95% CI]: 0.85 [0.82–0.87]) and Asian/Pacific islander (IRR [95% CI]: 0.94 [0.89–0.98]). In addition, beneficiaries enrolled in the Medicaid program (a proxy of low-income) were 8% less likely to initiate a newer GLD (IRR [95% CI]: 0.92 [0.90–0.94]). Individuals living in urban areas were slightly more likely to initiate a newer GLD than those living in rural areas (IRR [95% CI]: 1.03 [1.01–1.05]). Beneficiaries with CKD were 66% more likely to initiate a newer GLD (IRR [95% CI]: 1.66 [1.63–1.70]), whereas those with CVD were 21% less likely to initiate a newer GLD (IRR [95% CI]: 0.79 [0.77–0.81]), compared to their counterparts.

**Table 2:** Factors associated with newer GLD initiation among Medicare beneficiaries with type 2 diabetes.

Variable	IRR	95% CI	*P*
**Age (per 10-year change)**	0.69	0.69–0.70	<.001
**Sex**			
Male	Ref		
Female	0.99	0.97–1.01	0.222
**Race/ethnicity**			
Non-Hispanic White	Ref		
Non-Hispanic Black	0.66	0.64–0.68	<.001
Hispanic	0.85	0.82–0.87	<.001
Asian/Pacific islander	0.94	0.89–0.98	0.004
American Indian/Alaska Native	0.74	0.66–0.82	<.001
Other	1.16	1.10–1.21	<.001
**Medicaid dual eligibility**			
No	Ref		
Yes	0.92	0.90–0.94	<.001
**Rural/urban classification**			
Rural	Ref		
Urban	1.03	1.01–1.05	0.006
**T2D duration**	1.01	1.01–1.02	<.001
**Number of GLD classes prescribed**	1.61	1.59–1.64	<.001
T2D duration*Number of GLD classes prescribed	0.99	0.99–0.99	<.001
**Cardiovascular disease**			
No	Ref		
Yes	0.79	0.77–0.81	<.001
**Chronic kidney disease**			
No	Ref		
Yes	1.66	1.63–1.70	<.001

IRR: initiation rate ratio; Ref: referent; GLD: glucose-lowering drug; T2D: type 2 diabetes; CI: confidence interval; *P*: *p*-value.

**Fig 1:**
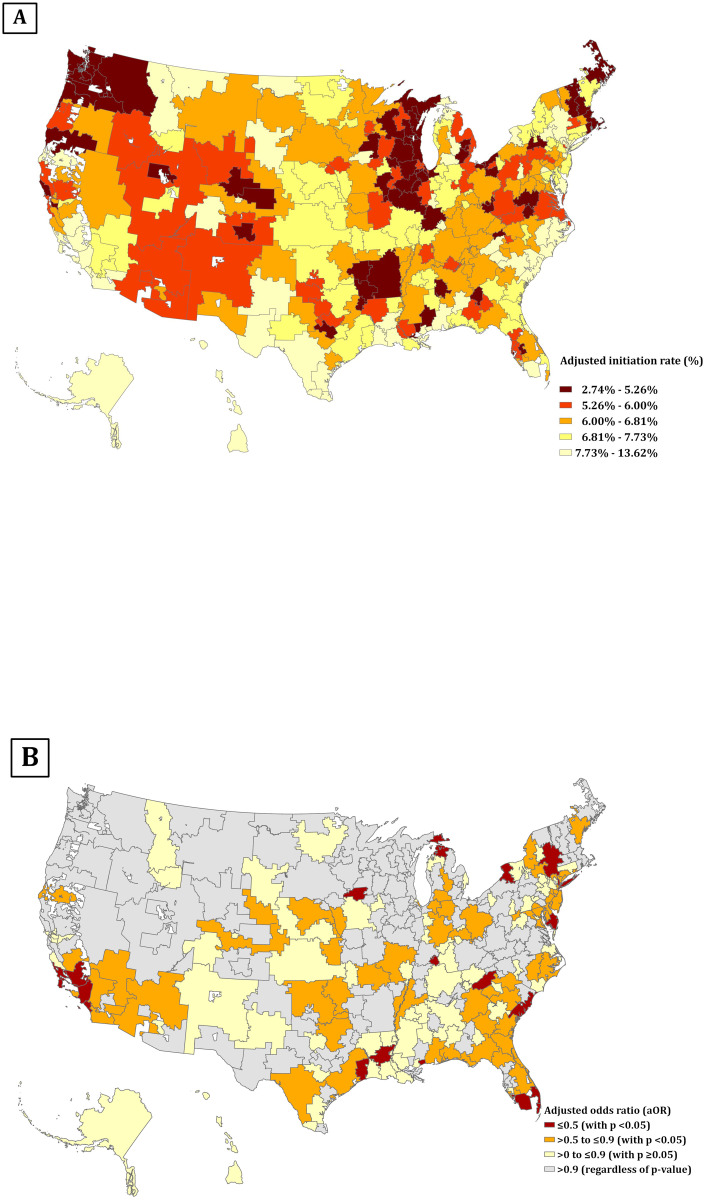
Adjusted initiation rate of newer GLDs and racial disparities in the initiation among Medicare beneficiaries with T2D in the entire US, by HRR. (A) Adjusted initiation rate of newer GLDs among Medicare beneficiaries with T2D, by HRR; the initiation rate was calculated using multivariable Poisson regression model, adjusting for age, sex, Medicaid dual eligibility, rural/urban classification, T2D duration, number of GLD classes prescribed, CVD, and CKD. Each color corresponds to quintiles of adjusted initiation rate, ranging from 2.7% to 13.6%. (B) Racial disparities in the initiation of newer GLDs among Medicare beneficiaries with T2D, by HRR; the racial disparities in the initiation of newer GLDs were determined using multivariable logistic regression model, adjusting for age, sex, Medicaid dual eligibility, rural/urban classification, T2D duration, number of GLD classes prescribed, CVD, and CKD. The dark red color stands for HRRs with marked disparities in the initiation of GLDs that Black people were >50% less likely to initiate the newer GLDs compared to White people (n = 22 HRRs); the orange color stands for HRRs with disparities in the initiation of GLDs that Black people were 10–50% less likely to initiate the newer GLDs compared to White people (n = 57 HRRs); the yellow color stands for HRRs appeared to have disparities in the initiation of GLDs but without satanical significance (aOR <0.9 and *p*>.05); the grey areas stand for HRRs with negligible racial disparities in the initiation of GLDs.

In the 15% sample of national Medicare fee-for-service beneficiaries, we observed significant geographic variation in the initiation of newer GLDs across HRRs (Fig 1A), with an adjusted initiation rate spanning 2.7%-13.6%. The 30 HRRs with the lowest initiation rate were concentrated in Wisconsin (n = 5) and Illinois (n = 4). On the other hand, the 30 HRRs with the highest initiation rate were concentrated in California (n = 6) and New York (n = 4). Moreover, we identified a significant geographic variation in racial disparities in initiating a newer GLD. Of 306 HRRs (Fig 1B), 22 were identified to have marked racial disparities in which non-Hispanic Black individuals were ≥50% less likely to initiate a newer GLD than non-Hispanic White patients (aOR, ≤0.5; *p* < .05), concentrating on California (n = 5), New York (n = 4) and Florida (n = 3). In another 57 HRRs, non-Hispanic Black individuals were 10–50% less likely to initiate a newer GLD than non-Hispanic White individuals (aOR, >0.5 to ≤0.9; *p* <0.05). Collectively, HRRs with greater disparities in the initiation of newer GLDs were those regions with higher initiation rates.

**Fig 2:**
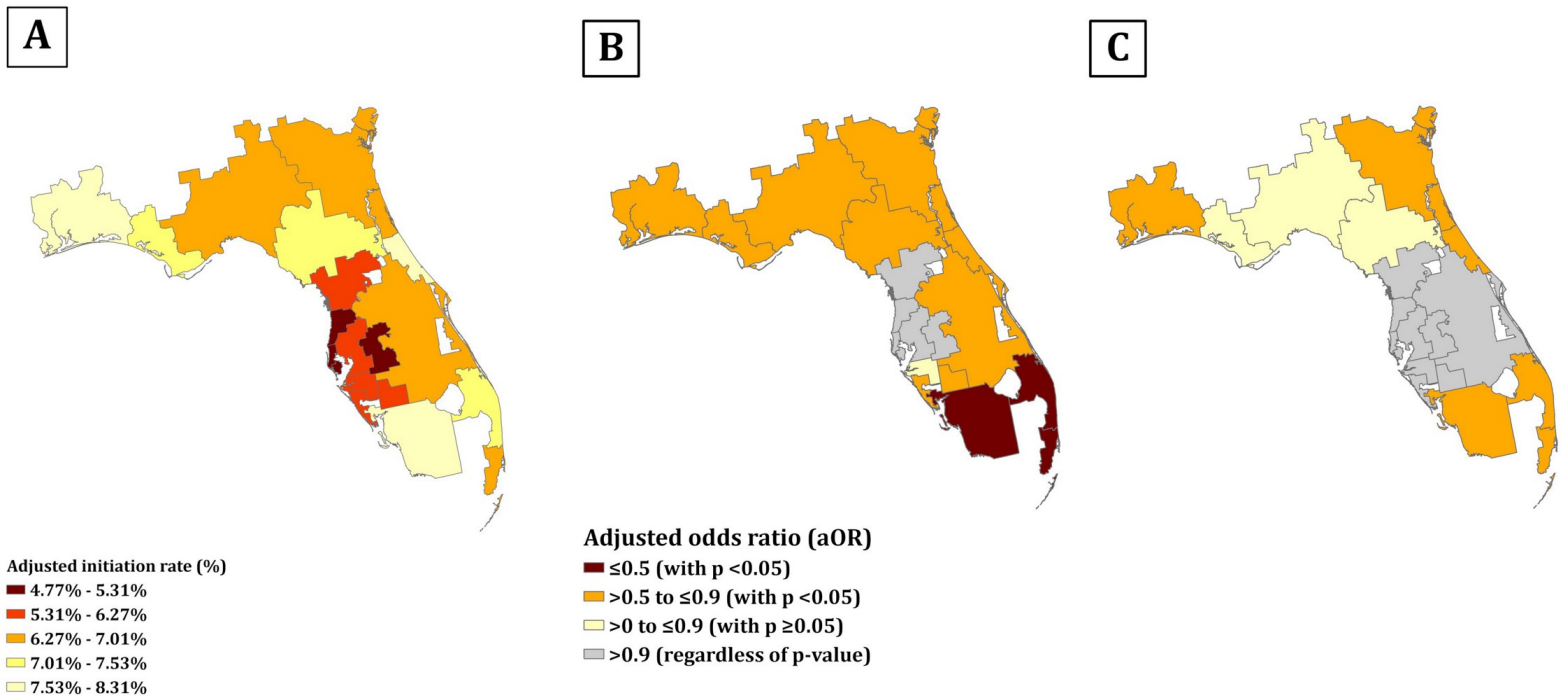
Adjusted initiation rate of newer GLDs and racial disparities in the initiation among Medicare beneficiaries with T2D in Florida state, by HRR. (A) Adjusted initiation rate of newer GLDs among Medicare beneficiaries with T2D in Florida state, by HRR; each color corresponds to quintiles of adjusted initiation rate, ranging from 4.8% to 8.3%.(B) (C) Racial disparities in the initiation of newer GLDs among Medicare beneficiaries with T2D in Florida state, by HRR; the racial disparities in the initiation of newer GLDs were determined using multivariable logistic regression model, adjusting for age, sex, Medicaid dual eligibility, rural/urban classification, T2D duration, number of GLD classes prescribed, CVD, and CKD. (B) presents the racial disparities between non-Hispanic Black vs. non-Hispanic White (referent); (C) presents the racial disparities between Hispanic vs. non-Hispanic White (referent).

Among the 100% samples of Florida Medicare fee-for-service beneficiaries, the adjusted initiation rate of newer GLD ranged from 4.8% to 8.3% (Fig 2A). Among the 18 HRRs in Florida, those located in Central Florida, e.g., St. Petersburg and Lakeland, had the lowest initiation rate; while Fort Myers and Ormond Beach, locating in South and Central East Florida, had the highest initiation rate. We identified the geographic variation in racial disparities in initiating a newer GLD among non-Hispanic Black (Fig 2B) and Hispanic patients (Fig 2C) compared to non-Hispanic White patients. Moreover, disparities in initiating a newer GLD for Black individuals were more prevalent than that seen in Hispanic individuals across Florida. Concordant to our national maps, HRRs with more distinct racial disparities were those regions with higher initiation rates in Florida.

## Discussion

In a nationally representative sample of older population with T2D who are being treated with GLDs, we found that initiation of newer GLDs with cardiovascular benefits remained low. Although the ideal initiation rate of newer GLDs is unidentified in previous research, we suggest that older adults remain in dire needs for better treatment given the high percentage of CVD and renal comorbidities in this population. The observed racial disparities of initiation rates in this study underscore the urgent need to ensure equitable access to these newer cardioprotective treatments for underrepresented groups with T2D. Older age individuals, racial and ethnic minority groups, Medicaid enrollees, and rural residents were less likely to initiate newer GLDs. Furthermore, we identified significant geographic variation in the initiation of the newer GLDs across the nation, as well as geographic variation in racial disparities in initiating these newer GLDs. Regions with higher initiation rates of newer GLDs were those regions with more remarkable disparities across racial and ethnic groups, showing that newer GLDs may have been utilized by those with higher socioeconomic status, which contributed to higher disparities.

Within both 15% national samples and 100% Florida samples, we not only characterized the geographic distribution of newer GLD initiation but also pinpointed the areas with the most salient racial and ethnic disparities. Strikingly, we have observed an inverse correlation of geographic distribution between the initiation rate of newer GLD use and its racial disparities. That is, our data have demonstrated that regions with higher initiation rates of newer GLDs were those regions with more remarkable disparities across racial and ethnic groups. However, we also observed that the number of non-Hispanic Black patients were extremely low in some HRRs. This may result in aOR with wider confidence intervals or insignificant results due to underrepresented Black patients. Numbers of population and initiations by race in each HRR and corresponding aORs, 95% CIs, and p-values are recorded in S1 Table. Nevertheless, these results have still shown that racial and ethnic groups and those at increased risk population groups are not benefiting from those novel treatments, and that, the advance in medical therapies and technologies has the potential to exacerbate the existing disparities. A previous study indicated that the inadequate initiation of newer GLD among racial and ethnic minority groups may widen the existing gap in T2D care.[18] Our study supports a spatially explicit data-driven approach in developing interventions for addressing disparities in the adoption of evidence-based T2D treatment. Therefore, our study may shed light on health policy interventions by identifying HRRs with the most inequitable health access so that policymakers and stakeholders could allocate resources precisely.

Our observations were consistent with previous studies suggesting under-utilization of newer GLDs in the US, especially those at an older age and increased cardiovascular risks, despite the proven cardiovascular benefits of these newer therapies.[6,19] Racial and ethnic disparities in T2D care, treatment and health outcomes in the Black population have been long recognized, including the uptake of these newer GLDs with cardiorenal benefits.[20] Studies using commercial insurance claims data showed that Black and Hispanic patients were 50% and 34% less likely to initiate SGLT2i, and 39% and 41% less likely to initiate GLP1a than White patients.[9,21,22] A post hoc analysis of the Look Ahead clinical trial first examined that Black and American Indian/Alaska Native individuals, who carry the highest risk of T2D and CVD across all racial and ethnic groups,[2,23] were 19% and 49% less likely to initiate newer diabetes medications, respectively, compared to non-Hispanic White patients.[18] However, secondary analysis from clinical trials with a small sample size limits the generalizability and thus calls for real-world studies. Different from most past studies which either focused on some specific subgroups or used a small sample size, our study has observed markedly significant results among other racial/ethnic minority groups simultaneously, including American Indian/Alaska Native, Hispanic, Asian/Pacific islander groups, with 26%, 15%, 6% lower initiation rate, respectively. Our results have ascertained previous studies on the under-utilization of newer GLDs among non-Hispanic Black patients and highlighted the racial and ethnic disparities in the newer treatment uptake in T2D beyond the non-Hispanic Black group in a nationally representative cohort.

In addition to race and ethnicity, we identified key clinical and sociodemographic factors associated with the initiation of newer GLDs. Although Medicaid expansion programs reduced the out-of-pocket costs of these newer GLDs,[24] beneficiaries with dual Medicare and Medicaid enrollment, those experiencing most socioeconomic disadvantages, still had lower rates of initiating these newer and outcome-improving therapies. Previous studies found that Medicaid expansion programs were associated with better cardiovascular outcomes, greater access to out-patient care, and eventually higher uptake of newer GLDs among patients with low income.[25,26] However, Medicaid expansion programs were not implemented consistently across states. Thus, at a national level, we observed dual Medicare and Medicaid eligibility as a low-income proxy (negative factor) instead of a contributory driver to newer GLD initiation.

Our study has several limitations. First, using claims data, we collected information on prescription fills. Thus, we could only know whether a patient filled their prescription but did not have information on patients’ behaviors on using the medication. Secondly, the enrollment file, as well as medical and drug claims in Medicare data, provided us with data on patients’ sociodemographics, comorbidities, and comedications. However, we could not obtain essential clinical information such as body mass index and hemoglobin A1c, as well as patient preference toward different treatments. Consequently, unmeasured confounding factors may lie in our study. The observed racial disparities in newer GLD initiation might be associated with multifaceted social determinants of health, which are other underlying factors not examined in this study, e.g., education level, housing stability, and food insecurity.[27] For instance, patients living in neighborhoods with higher education levels and socioeconomic status had higher chance of receiving newer GLD.[28] Previous studies also found that racial disparities in the distribution of SDOH could result in uneven access to T2D care, treatment, and health outcomes among different race/ethnicity.[29] Future studies are needed to examine these underlying social barriers to accessing newer treatments. Our study may also involve race/ethnicity misclassification issues since the RTI imputation algorithm is not self-reported. However, previous validation studies reported a high specificity (99%) of the RTI race/ethnicity variable.[30] Also, since our analyses were conducted in Medicare fee-for-service beneficiaries that only included the older population (>65 years), our results may not be generalizable to the younger population and those with private insurance. Another limitation lies in the study power. Among those HRRs with small populations, we may not have adequate statistical power to detect the disparities. Finally, in our cohort of Medicare beneficiaries with T2D who were treated with GLDs, we characterized the geographic variation in the uptake of newer treatment with cardiovascular benefits across entire US HRRs. Furthermore, we documented geographic variation and the racial disparities in initiating these newer treatments.

In conclusion, racial disparities are prevalent and striking in nearly half of HRRs across the nation. Regions with higher initiation rates of newer GLDs were those regions with more remarkable disparities across racial and ethnic groups. Moreover, we substantiated that cardioprotective newer GLDs were underutilized among Medicare beneficiaries with T2D, who were at high CVD risks and in immediate need of equitable access to those newer treatments. Our study highlights the importance of using an explicit data-driven approach to address disparities in the health care of T2D in the US. Social determinants of health are thought to be the root cause racial disparities. Diabetes is a public health crisis that must be managed not only by traditional medical care but also by addressing patients’ unmet social needs. There is a critical need to integrate social care into health care of diabetes. Future studies are needed to understand the underlying causes of such structural inequities in T2D health care and to develop interventions to improve equitable access to evidence-based therapies among millions of Americans with T2D.

## Supporting information

S1 Table Number of beneficiaries initiated newer GLD per HRR by race/ethnicity. (DOCX)

## Abbreviations

GLDs: glucose-lowering drugs
SGLT2i: sodium-glucose cotransporter-2 inhibitors
GLP1a: glucagon-like peptide-1 receptor agonists
T2D: type 2 diabetes
HRRs: hospital-referral regions
CVD: cardiovascular disease
CKD: chronic kidney disease
RTI: Research Triangle Institute
RUCC: Rural-Urban Continuum Codes
SD: standard deviation
IQR: interquartile range
aOR: adjusted odds ratio
IRR: initiation rate ratio
