# Supplementary material for: Geographic variation and racial disparities in adoption of newer glucose-lowering drugs with cardiovascular benefits among US Medicare beneficiaries with type 2 diabetes
Source: PLoS One. 2024 Jan 29;19(1):e0297208. doi: 10.1371/journal.pone.0297208 (PMC10824445; doi:10.1371/journal.pone.0297208)
Supplement: S1 Table — (DOCX) [file pone.0297208.s001.docx]

**Supplemental Table 1.** Number of beneficiaries initiated newer GLD per HRR by race/ethnicity

| City | State | Total population per HRR | Total number of Black per HRR | Number of Black initiated | Total number of White per HRR | Number of White initiated | Total number of Hispanic per HRR | Number of Hispanic initiated |
| --- | --- | --- | --- | --- | --- | --- | --- | --- |
| Birmingham | AL | 8679 | 1125 | 64 | 3750 | 280 | 44 | * |
| Dothan | AL | 3839 | 512 | 29 | 1699 | 119 | 24 | * |
| Huntsville | AL | 2512 | 242 | 13 | 1119 | 66 | * | * |
| Mobile | AL | 2599 | 408 | 27 | 1107 | 103 | 18 | * |
| Montgomery | AL | 1233 | 298 | 18 | 385 | 34 | * | * |
| Tuscaloosa | AL | 1183 | 330 | 14 | 363 | 21 | * | * |
| Anchorage | AK | 1394 | 26 | * | 567 | 45 | 61 | * |
| Mesa | AZ | 3103 | 56 | * | 1369 | 103 | 168 | * |
| Phoenix | AZ | 9080 | 177 | * | 3665 | 258 | 837 | 36 |
| Sun City | AZ | 1358 | 19 | * | 605 | 37 | 38 | * |
| Tucson | AZ | 2633 | 34 | * | 1068 | 77 | 225 | * |
| Fort Smith | AR | 1675 | 26 | * | 890 | 56 | 27 | * |
| Jonesboro | AR | 1238 | 34 | * | 739 | 60 | 11 | * |
| Little Rock | AR | 6946 | 708 | 26 | 3424 | 208 | 50 | * |
| Springdale | AR | 1623 | * | * | 905 | 68 | 59 | * |
| Texarkana | AR | 1270 | 184 | * | 560 | 34 | 13 | * |
| Orange County | CA | 8595 | 55 | * | 1853 | 185 | 635 | 54 |
| Bakersfield | CA | 4392 | 76 | * | 942 | 96 | 1176 | 124 |
| Chico | CA | 1902 | 17 | * | 825 | 63 | 129 | * |
| Contra Costa County | CA | 2339 | 123 | * | 587 | 45 | 269 | 12 |
| Fresno | CA | 4712 | 113 | * | 961 | 83 | 1223 | 108 |
| Los Angeles | CA | 30689 | 1180 | 57 | 5414 | 563 | 4138 | 314 |
| Modesto | CA | 3632 | 65 | * | 990 | 73 | 785 | 51 |
| Napa | CA | 1303 | 13 | * | 518 | 32 | 135 | * |
| Alameda County | CA | 4157 | 390 | * | 577 | 30 | 409 | 17 |
| Palm Springs/Rancho Mira | CA | 1444 | 17 | * | 464 | 29 | 115 | 12 |
| Redding | CA | 2061 | 11 | * | 928 | 83 | 59 | * |
| Sacramento | CA | 7550 | 249 | 16 | 2451 | 149 | 754 | 44 |
| Salinas | CA | 1706 | 23 | * | 317 | 28 | 511 | 27 |
| San Bernardino | CA | 4871 | 258 | 17 | 1008 | 91 | 925 | 68 |
| San Diego | CA | 7500 | 190 | 15 | 1771 | 157 | 1574 | 174 |
| San Francisco | CA | 4597 | 271 | 18 | 791 | 52 | 416 | 17 |
| San Jose | CA | 4539 | 62 | * | 800 | 49 | 527 | 33 |
| San Luis Obispo | CA | 1305 | * | * | 524 | 49 | 99 | * |
| San Mateo County | CA | 1705 | 26 | * | 465 | 28 | 165 | * |
| Santa Barbara | CA | 1905 | 25 | * | 487 | 61 | 380 | 55 |
| Santa Cruz | CA | 1119 | * | * | 320 | 17 | 256 | 16 |
| Santa Rosa | CA | 1250 | * | * | 449 | 26 | 133 | * |
| Stockton | CA | 1928 | 88 | * | 434 | 35 | 381 | 22 |
| Ventura | CA | 3128 | 27 | * | 815 | 71 | 583 | 56 |
| Boulder | CO | 468 | * | * | 209 | 23 | 35 | * |
| Colorado Springs | CO | 1831 | 25 | * | 798 | 50 | 247 | 14 |
| Denver | CO | 4329 | 139 | * | 1909 | 149 | 411 | 20 |
| Fort Collins | CO | 911 | * | * | 488 | 27 | 60 | * |
| Grand Junction | CO | 793 | * | * | 425 | 38 | 64 | * |
| Greeley | CO | 1095 | * | * | 533 | 24 | 149 | * |
| Pueblo | CO | 672 | * | * | 244 | 12 | 161 | 11 |
| Bridgeport | CT | 2703 | 183 | 13 | 833 | 64 | 190 | 20 |
| Hartford | CT | 5052 | 224 | 19 | 1975 | 145 | 267 | 26 |
| New Haven | CT | 5863 | 299 | 21 | 2155 | 170 | 323 | 18 |
| Wilmington | DE | 5079 | 549 | 38 | 1705 | 136 | 118 | * |
| Washington | DC | 10074 | 1831 | 89 | 2847 | 199 | 249 | 12 |
| Bradenton | FL | 8927 | 275 | * | 3539 | 222 | 282 | 12 |
| Clearwater | FL | 10882 | 199 | * | 4316 | 234 | 254 | 14 |
| Fort Lauderdale | FL | 79065 | 3747 | 162 | 23036 | 1736 | 2790 | 119 |
| Fort Myers | FL | 44291 | 1051 | 54 | 17030 | 1408 | 2398 | 199 |
| Gainesville | FL | 15132 | 1367 | 84 | 6140 | 489 | 347 | 32 |
| Hudson | FL | 11581 | 185 | * | 4602 | 282 | 388 | 19 |
| Jacksonville | FL | 29627 | 2997 | 146 | 11160 | 905 | 617 | 44 |
| Lakeland | FL | 7549 | 433 | 18 | 2974 | 178 | 358 | 14 |
| Miami | FL | 57906 | 2965 | 115 | 6428 | 466 | 15604 | 800 |
| Ocala | FL | 23850 | 712 | 38 | 9798 | 587 | 601 | 33 |
| Orlando | FL | 87420 | 4940 | 252 | 31069 | 2118 | 5046 | 270 |
| Ormond Beach | FL | 11286 | 507 | 42 | 4405 | 352 | 235 | 20 |
| Panama City | FL | 5997 | 383 | 27 | 2451 | 204 | 50 | * |
| Pensacola | FL | 13949 | 1117 | 72 | 6105 | 532 | 171 | 17 |
| Sarasota | FL | 18208 | 326 | 11 | 7508 | 474 | 352 | 15 |
| St. Petersburg | FL | 8991 | 632 | 14 | 3113 | 172 | 228 | * |
| Tallahassee | FL | 8516 | 1601 | 72 | 2944 | 230 | 127 | * |
| Tampa | FL | 22802 | 1494 | 97 | 7037 | 417 | 2444 | 149 |
| Albany | GA | 702 | 160 | * | 253 | 19 | * | * |
| Atlanta | GA | 16708 | 1925 | 90 | 6624 | 460 | 343 | 26 |
| Augusta | GA | 2316 | 475 | 27 | 857 | 64 | 25 | * |
| Columbus | GA | 1075 | 221 | * | 333 | 20 | * | * |
| Macon | GA | 2609 | 524 | 28 | 828 | 74 | 11 | * |
| Rome | GA | 1382 | 67 | * | 740 | 66 | 15 | * |
| Savannah | GA | 2954 | 378 | 21 | 1232 | 98 | 29 | * |
| Honolulu | HI | 3265 | 12 | * | 312 | 26 | 120 | * |
| Boise | ID | 2117 | * | * | 1133 | 73 | 150 | 12 |
| Idaho Falls | ID | 877 | * | * | 514 | 42 | 42 | * |
| Aurora | IL | 910 | 43 | * | 383 | 17 | 122 | 11 |
| Blue Island | IL | 3611 | 564 | 17 | 1206 | 56 | 184 | * |
| Chicago | IL | 8465 | 1724 | 58 | 1241 | 68 | 1101 | 41 |
| Elgin | IL | 2874 | 47 | * | 1275 | 81 | 168 | * |
| Evanston | IL | 3966 | 62 | * | 1676 | 116 | 118 | * |
| Hinsdale | IL | 1553 | 57 | * | 625 | 31 | 52 | * |
| Joliet | IL | 2643 | 124 | * | 1203 | 75 | 128 | * |
| Melrose Park | IL | 4683 | 239 | 11 | 1803 | 104 | 451 | 28 |
| Peoria | IL | 2378 | 73 | * | 1354 | 74 | 41 | * |
| Rockford | IL | 3126 | 115 | * | 1624 | 101 | 129 | * |
| Springfield | IL | 4194 | 137 | * | 2476 | 140 | 13 | * |
| Urbana | IL | 1583 | 65 | * | 911 | 64 | 26 | * |
| Bloomington | IL | 605 | 12 | * | 364 | 17 | * | * |
| Evansville | IN | 3475 | 70 | * | 2087 | 125 | 14 | * |
| Fort Wayne | IN | 2987 | 97 | * | 1706 | 159 | 58 | * |
| Gary | IN | 2414 | 223 | * | 1065 | 71 | 95 | * |
| Indianapolis | IN | 11973 | 743 | 44 | 6348 | 571 | 110 | * |
| Lafayette | IN | 974 | 13 | * | 573 | 46 | 15 | * |
| Muncie | IN | 968 | 36 | * | 539 | 37 | * | * |
| Munster | IN | 1519 | 152 | * | 507 | 34 | 126 | 14 |
| South Bend | IN | 2568 | 117 | * | 1434 | 131 | 54 | * |
| Terre Haute | IN | 1020 | 23 | * | 602 | 56 | * | * |
| Cedar Rapids | IA | 1218 | 17 | * | 766 | 67 | * | * |
| Davenport | IA | 1904 | 65 | * | 1051 | 74 | 35 | * |
| Des Moines | IA | 4641 | 70 | * | 2818 | 224 | 59 | * |
| Dubuque | IA | 404 | * | * | 221 | * | * | * |
| Iowa City | IA | 1413 | 20 | * | 855 | 40 | 21 | * |
| Mason City | IA | 826 | * | * | 517 | 69 | * | * |
| Sioux City | IA | 1209 | * | * | 716 | 45 | 29 | * |
| Waterloo | IA | 930 | 35 | * | 537 | 35 | * | * |
| Topeka | KS | 1917 | 50 | * | 1088 | 104 | 47 | * |
| Wichita | KS | 6208 | 164 | 14 | 3526 | 275 | 238 | 16 |
| Covington | KY | 1123 | * | * | 693 | 64 | * | * |
| Lexington | KY | 7992 | 160 | * | 4475 | 382 | 34 | * |
| Louisville | KY | 7274 | 377 | 26 | 3718 | 299 | 32 | * |
| Owensboro | KY | 859 | 17 | * | 476 | 61 | * | * |
| Paducah | KY | 2193 | 88 | 15 | 1241 | 105 | * | * |
| Alexandria | LA | 1719 | 273 | 19 | 678 | 91 | 14 | * |
| Baton Rouge | LA | 2606 | 646 | 33 | 848 | 58 | 21 | * |
| Houma | LA | 1232 | 150 | * | 580 | 51 | 18 | * |
| Lafayette | LA | 3246 | 611 | 52 | 1227 | 109 | 41 | * |
| Lake Charles | LA | 1306 | 164 | 15 | 572 | 60 | 16 | * |
| Metairie | LA | 1214 | 153 | * | 489 | 30 | 31 | * |
| Monroe | LA | 1435 | 275 | 16 | 533 | 44 | * | * |
| New Orleans | LA | 1585 | 461 | 37 | 274 | 23 | 47 | * |
| Shreveport | LA | 2992 | 676 | 27 | 1035 | 68 | 21 | * |
| Slidell | LA | 630 | 59 | * | 246 | 27 | * | * |
| Bangor | ME | 2381 | * | * | 1433 | 93 | * | * |
| Portland | ME | 4055 | 21 | * | 2238 | 195 | 19 | * |
| Baltimore | MD | 12913 | 2174 | 132 | 3917 | 316 | 120 | * |
| Salisbury | MD | 3715 | 383 | 31 | 1607 | 168 | 30 | * |
| Takoma Park | MD | 3267 | 616 | 33 | 600 | 48 | 239 | 17 |
| Boston | MA | 19711 | 826 | 58 | 7913 | 438 | 1083 | 87 |
| Springfield | MA | 3272 | 128 | * | 1367 | 84 | 303 | 26 |
| Worcester | MA | 2753 | 52 | * | 1272 | 98 | 181 | 27 |
| Ann Arbor | MI | 5322 | 391 | 16 | 2060 | 108 | 64 | * |
| Dearborn | MI | 2765 | 101 | * | 1014 | 56 | 73 | * |
| Detroit | MI | 8655 | 1581 | 78 | 2015 | 118 | 98 | * |
| Flint | MI | 2877 | 260 | * | 949 | 36 | 28 | * |
| Grand Rapids | MI | 3017 | 169 | 16 | 1516 | 116 | 113 | * |
| Kalamazoo | MI | 2478 | 121 | * | 1321 | 129 | 25 | * |
| Lansing | MI | 2808 | 116 | * | 1481 | 140 | 58 | * |
| Marquette | MI | 791 | * | * | 465 | 16 | * | * |
| Muskegon | MI | 1016 | 79 | * | 464 | 39 | 20 | * |
| Petoskey | MI | 995 | * | * | 512 | 63 | * | * |
| Pontiac | MI | 2100 | 101 | * | 739 | 40 | 37 | * |
| Royal Oak | MI | 3914 | 320 | 12 | 1152 | 59 | 29 | * |
| Saginaw | MI | 3760 | 124 | * | 1823 | 128 | 73 | * |
| St. Joseph | MI | 643 | 61 | * | 301 | 23 | 18 | * |
| Traverse City | MI | 1263 | * | * | 708 | 59 | * | * |
| Duluth | MN | 826 | * | * | 477 | 37 | * | * |
| Minneapolis | MN | 4930 | 264 | 29 | 2631 | 219 | 77 | * |
| Rochester | MN | 885 | * | * | 511 | 19 | 20 | * |
| St. Cloud | MN | 329 | 11 | * | 189 | 20 | * | * |
| St. Paul | MN | 1621 | 118 | * | 790 | 65 | 46 | * |
| Gulfport | MS | 710 | 99 | * | 273 | 35 | * | * |
| Hattiesburg | MS | 1630 | 315 | 15 | 747 | 48 | * | * |
| Jackson | MS | 5504 | 1657 | 108 | 1738 | 121 | 17 | * |
| Meridian | MS | 1228 | 380 | 26 | 404 | 34 | * | * |
| Oxford | MS | 994 | 231 | 14 | 380 | 33 | * | * |
| Tupelo | MS | 2655 | 513 | 38 | 1241 | 108 | * | * |
| Cape Girardeau | MO | 1617 | 87 | * | 868 | 77 | * | * |
| Columbia | MO | 3346 | 98 | * | 1942 | 142 | 17 | * |
| Joplin | MO | 1846 | 11 | * | 1006 | 94 | 11 | * |
| Kansas City | MO | 7926 | 438 | 27 | 4019 | 285 | 124 | * |
| Springfield | MO | 3088 | 14 | * | 1829 | 174 | 31 | * |
| St. Louis | MO | 12562 | 1095 | 74 | 5919 | 497 | 77 | * |
| Billings | MT | 2051 | * | * | 1238 | 84 | 40 | * |
| Great Falls | MT | 528 | * | * | 284 | 25 | * | * |
| Missoula | MT | 1354 | * | * | 814 | 71 | 18 | * |
| Lincoln | NE | 2675 | 18 | * | 1649 | 136 | 45 | * |
| Omaha | NE | 5023 | 135 | * | 2937 | 236 | 118 | 24 |
| Las Vegas | NV | 5813 | 327 | 21 | 1727 | 141 | 578 | 40 |
| Reno | NV | 2518 | 25 | * | 1154 | 80 | 161 | * |
| Lebanon | NH | 2105 | * | * | 1205 | 62 | 13 | * |
| Manchester | NH | 3730 | 11 | * | 2023 | 109 | 43 | * |
| Camden | NJ | 19104 | 863 | 53 | 6235 | 451 | 593 | 37 |
| Hackensack | NJ | 7610 | 198 | * | 1771 | 137 | 633 | 52 |
| Morristown | NJ | 4890 | 146 | * | 1675 | 126 | 142 | * |
| New Brunswick | NJ | 5540 | 229 | 16 | 1504 | 119 | 218 | 26 |
| Newark | NJ | 6693 | 844 | 38 | 1324 | 99 | 521 | 49 |
| Paterson | NJ | 2022 | 96 | * | 590 | 32 | 159 | 11 |
| Ridgewood | NJ | 2563 | 89 | * | 826 | 65 | 59 | * |
| Albuquerque | NM | 5037 | 41 | * | 1213 | 90 | 1141 | 73 |
| Albany | NY | 7847 | 269 | 14 | 3161 | 293 | 156 | 15 |
| Binghamton | NY | 1564 | 23 | * | 813 | 77 | 14 | * |
| Bronx | NY | 3640 | 580 | 28 | 377 | 24 | 636 | 43 |
| Buffalo | NY | 3260 | 265 | 23 | 1356 | 165 | 106 | * |
| Elmira | NY | 1301 | 36 | * | 641 | 61 | 24 | * |
| East Long Island | NY | 28870 | 1216 | 79 | 6973 | 605 | 902 | 63 |
| Manhattan | NY | 23074 | 1828 | 62 | 4748 | 447 | 1465 | 93 |
| Rochester | NY | 2914 | 220 | 19 | 1231 | 113 | 120 | * |
| Syracuse | NY | 3348 | 75 | * | 1703 | 141 | 38 | * |
| White Plains | NY | 5891 | 359 | 24 | 1640 | 137 | 269 | 19 |
| Asheville | NC | 3275 | 69 | 11 | 1804 | 226 | 25 | * |
| Charlotte | NC | 9276 | 1113 | 65 | 4002 | 295 | 173 | * |
| Durham | NC | 6142 | 1319 | 89 | 2078 | 139 | 79 | * |
| Greensboro | NC | 1669 | 247 | 18 | 679 | 63 | 20 | * |
| Greenville | NC | 4919 | 1240 | 91 | 1749 | 168 | 39 | * |
| Hickory | NC | 1399 | 52 | * | 743 | 46 | 18 | * |
| Raleigh | NC | 8079 | 1606 | 153 | 2691 | 269 | 161 | 16 |
| Wilmington | NC | 2979 | 392 | 40 | 1228 | 118 | 20 | * |
| Winston-Salem | NC | 3927 | 295 | 22 | 1891 | 131 | 58 | * |
| Bismarck | ND | 892 | * | * | 537 | 36 | * | * |
| Fargo/Moorhead MN | ND | 1547 | * | * | 916 | 81 | 17 | * |
| Grand Forks | ND | 553 | * | * | 322 | 17 | 11 | * |
| Minot | ND | 530 | * | * | 307 | 26 | * | * |
| Akron | OH | 1737 | 126 | * | 841 | 61 | * | * |
| Canton | OH | 1783 | 48 | * | 1017 | 74 | * | * |
| Cincinnati | OH | 4900 | 396 | 19 | 2471 | 201 | 17 | * |
| Cleveland | OH | 6798 | 721 | 23 | 2924 | 157 | 105 | 11 |
| Columbus | OH | 10665 | 492 | 30 | 5769 | 522 | 52 | * |
| Dayton | OH | 3624 | 237 | * | 1890 | 141 | 16 | * |
| Elyria | OH | 952 | 41 | * | 496 | 30 | 45 | * |
| Kettering | OH | 1268 | 36 | * | 657 | 56 | * | * |
| Toledo | OH | 4238 | 201 | 11 | 2151 | 159 | 94 | * |
| Youngstown | OH | 2370 | 123 | 11 | 1115 | 94 | 27 | * |
| Lawton | OK | 890 | 57 | * | 390 | 31 | 39 | * |
| Oklahoma City | OK | 7560 | 362 | 28 | 3712 | 335 | 218 | 20 |
| Tulsa | OK | 5636 | 279 | 16 | 2610 | 225 | 83 | * |
| Bend | OR | 748 | * | * | 407 | 32 | 19 | * |
| Eugene | OR | 2124 | * | * | 1186 | 76 | 33 | * |
| Medford | OR | 1800 | * | * | 974 | 44 | 55 | * |
| Portland | OR | 4793 | 77 | * | 2454 | 127 | 226 | * |
| Salem | OR | 615 | * | * | 314 | 29 | 52 | * |
| Allentown | PA | 5867 | 116 | * | 2686 | 193 | 211 | 23 |
| Altoona | PA | 1048 | * | * | 627 | 40 | * | * |
| Danville | PA | 2183 | 22 | * | 1260 | 82 | 17 | * |
| Erie | PA | 2944 | 35 | * | 1679 | 124 | 20 | * |
| Harrisburg | PA | 3554 | 98 | * | 1865 | 150 | 86 | * |
| Johnstown | PA | 504 | * | * | 292 | 30 | * | * |
| Lancaster | PA | 2415 | 51 | * | 1215 | 84 | 68 | * |
| Philadelphia | PA | 16213 | 1712 | 72 | 5173 | 387 | 391 | 30 |
| Pittsburgh | PA | 6331 | 263 | 11 | 3402 | 235 | 23 | * |
| Reading | PA | 2547 | 58 | * | 1288 | 84 | 98 | * |
| Sayre | PA | 823 | * | * | 503 | 27 | * | * |
| Scranton | PA | 1909 | 20 | * | 952 | 81 | 43 | * |
| Wilkes-Barre | PA | 1225 | 11 | * | 611 | 45 | 23 | * |
| York | PA | 1734 | 37 | * | 911 | 89 | 31 | * |
| Providence | RI | 3752 | 98 | * | 1532 | 73 | 134 | * |
| Charleston | SC | 4814 | 700 | 43 | 2016 | 202 | 37 | * |
| Columbia | SC | 5494 | 1169 | 100 | 2017 | 197 | 27 | * |
| Florence | SC | 2445 | 648 | 58 | 863 | 71 | * | * |
| Greenville | SC | 3862 | 304 | 23 | 1872 | 165 | 26 | * |
| Spartanburg | SC | 1636 | 159 | 11 | 791 | 64 | * | * |
| Rapid City | SD | 723 | * | * | 397 | 39 | * | * |
| Sioux Falls | SD | 3072 | * | * | 1871 | 135 | 19 | * |
| Chattanooga | TN | 3143 | 151 | * | 1646 | 122 | * | * |
| Jackson | TN | 2075 | 197 | 15 | 1043 | 66 | * | * |
| Johnson City | TN | 952 | * | * | 543 | 30 | * | * |
| Kingsport | TN | 2362 | 19 | * | 1420 | 120 | * | * |
| Knoxville | TN | 5302 | 99 | * | 2979 | 233 | 24 | * |
| Memphis | TN | 7734 | 1797 | 98 | 2822 | 213 | 47 | * |
| Nashville | TN | 9911 | 566 | 44 | 5143 | 403 | 79 | * |
| Abilene | TX | 1250 | 25 | * | 578 | 45 | 151 | * |
| Amarillo | TX | 1453 | 42 | * | 678 | 49 | 204 | 11 |
| Austin | TX | 4043 | 183 | 11 | 1561 | 118 | 485 | 33 |
| Beaumont | TX | 1915 | 270 | * | 729 | 70 | 44 | * |
| Bryan | TX | 701 | 72 | * | 260 | 16 | 65 | * |
| Corpus Christi | TX | 1578 | 19 | * | 341 | 31 | 473 | 42 |
| Dallas | TX | 13749 | 1225 | 71 | 5158 | 408 | 930 | 72 |
| El Paso | TX | 2408 | 23 | * | 423 | 22 | 937 | 58 |
| Fort Worth | TX | 5255 | 361 | 13 | 2139 | 151 | 401 | 23 |
| Harlingen | TX | 2541 | * | * | 177 | 13 | 1260 | 143 |
| Houston | TX | 16153 | 1451 | 79 | 5045 | 443 | 1456 | 90 |
| Longview | TX | 841 | 94 | * | 364 | 17 | 27 | * |
| Lubbock | TX | 2673 | 60 | * | 903 | 74 | 615 | 56 |
| McAllen | TX | 2178 | * | * | 135 | 15 | 1105 | 155 |
| Odessa | TX | 1307 | 35 | * | 343 | 28 | 391 | 32 |
| San Angelo | TX | 702 | * | * | 222 | 18 | 164 | 14 |
| San Antonio | TX | 7566 | 159 | 21 | 1619 | 151 | 2392 | 192 |
| Temple | TX | 1047 | 103 | * | 360 | 20 | 144 | * |
| Tyler | TX | 2540 | 197 | 12 | 1166 | 79 | 89 | * |
| Victoria | TX | 902 | 40 | * | 280 | 17 | 192 | 15 |
| Waco | TX | 1101 | 108 | * | 504 | 32 | 89 | * |
| Wichita Falls | TX | 948 | 36 | * | 426 | 30 | 70 | * |
| Ogden | UT | 602 | * | * | 324 | 18 | 30 | * |
| Provo | UT | 1010 | * | * | 642 | 48 | 40 | * |
| Salt Lake City | UT | 4502 | 16 | * | 2540 | 151 | 219 | 11 |
| Burlington | VT | 3208 | 13 | * | 1775 | 129 | 16 | * |
| Arlington | VA | 4502 | 376 | 19 | 1287 | 84 | 278 | 20 |
| Charlottesville | VA | 2941 | 223 | * | 1544 | 89 | 20 | * |
| Lynchburg | VA | 1582 | 235 | * | 727 | 33 | * | * |
| Newport News | VA | 1932 | 332 | 18 | 732 | 45 | 15 | * |
| Norfolk | VA | 4300 | 889 | 47 | 1389 | 92 | 58 | * |
| Richmond | VA | 7197 | 1390 | 63 | 2660 | 155 | 58 | * |
| Roanoke | VA | 3943 | 193 | 14 | 2155 | 153 | 11 | * |
| Winchester | VA | 2070 | 67 | * | 1203 | 103 | 21 | * |
| Everett | WA | 1382 | * | * | 716 | 29 | 55 | * |
| Olympia | WA | 1166 | 12 | * | 624 | 34 | 25 | * |
| Seattle | WA | 6543 | 194 | * | 2831 | 173 | 196 | * |
| Spokane | WA | 5720 | 38 | * | 3085 | 191 | 318 | 15 |
| Tacoma | WA | 1633 | 57 | * | 700 | 39 | 44 | * |
| Yakima | WA | 1144 | * | * | 474 | 25 | 158 | * |
| Charleston | WV | 4720 | 95 | * | 2665 | 195 | * | * |
| Huntington | WV | 2039 | 23 | * | 1156 | 90 | * | * |
| Morgantown | WV | 1801 | 11 | * | 1055 | 81 | * | * |
| Appleton | WI | 649 | * | * | 379 | 13 | 14 | * |
| Green Bay | WI | 1452 | * | * | 818 | 44 | 16 | * |
| La Crosse | WI | 1162 | * | * | 731 | 55 | * | * |
| Madison | WI | 3464 | 83 | * | 2114 | 115 | 55 | * |
| Marshfield | WI | 1090 | * | * | 680 | 43 | * | * |
| Milwaukee | WI | 7409 | 520 | 24 | 3335 | 179 | 377 | 31 |
| Neenah | WI | 501 | * | * | 310 | 19 | * | * |
| Wausau | WI | 637 | * | * | 383 | 30 | * | * |
| Casper | WY | 792 | * | * | 472 | 28 | 27 | * |
